# Isolation and characterization of pharmaceutical grade human pentraxins, serum amyloid P component and C‐reactive protein, for clinical use

**DOI:** 10.1016/j.jim.2012.07.013

**Published:** 2012-10-31

**Authors:** Mark B. Pepys, J. Ruth Gallimore, Joanne Lloyd, Zhanhong Li, David Graham, Graham W. Taylor, Stephan Ellmerich, Palma P. Mangione, Glenys A. Tennent, Winston L. Hutchinson, David J. Millar, Gary Bennett, John More, David Evans, Yogesh Mistry, Stephen Poole, Philip N. Hawkins

**Affiliations:** a Wolfson Drug Discovery Unit, Centre for Amyloidosis and Acute Phase Proteins, Division of Medicine, University College London, Rowland Hill Street, London NW3 2PF, UK; b Bio Products Laboratory Ltd, Dagger Lane, Estree, Herts WD6 3BX, UK; c Scottish National Blood Transfusion Service, Castlelaw Building, Pentlands Science Park, Bushloan, Pennicuik, Midlothian EH26 0PZ, UK; d Department of Molecular Medicine, University of Pavia, Via Taramelli 3b, 27100 Pavia, Italy; e Pharmacy Department, Royal Free London NHS Foundation Trust, Pond Street, London NW3 2QG, UK; f National Institute for Biological Standards and Control, Blanche Lane, South Mimms, Potters Bar, Herts EN6 3QG, UK

**Keywords:** BVDV, bovine viral diarrhea virus, cGMP, current good manufacturing practice, CRP, C‐reactive protein, ELISA, enzyme linked immunosorbent assay, ESIMS, electrospray ionization mass spectrometry, HBsAg, hepatitis B surface antigen, HCV, hepatitis C virus, HIV, human immunodeficiency virus, IBRV, infectious bovine rhinotracheitis virus, IS, international standards, PBMCs, peripheral blood mononuclear cells, PBS, phosphate buffered saline, SAA, serum amyloid A protein, SAP, serum amyloid P component, C‐reactive protein, Cytokine, Good manufacturing practice, Mononuclear cells, Pharmaceutical, Serum amyloid P component

## Abstract

The human pentraxin proteins, serum amyloid P component (SAP) and C‐reactive protein (CRP) are important in routine clinical diagnosis, SAP for systemic amyloidosis and CRP for monitoring the non‐specific acute phase response. They are also targets for novel therapies currently in development but their roles in health and disease are controversial. Thus, both for clinical use and to rigorously elucidate their functions, structurally and functionally intact, pharmaceutical grade preparations of the natural, authentic proteins are required. We report here the production from normal human donor plasma and the characterization of the first such preparations. Importantly, we demonstrate that, contrary to reports using recombinant proteins and less well characterized preparations, neither CRP nor SAP stimulate the release by human peripheral blood mononuclear cells *in vitro* of any TNFα, IL‐6 or IL‐8, nor does SAP cause release of IL‐1β or IL‐10. Furthermore neither of our preparations was pro‐inflammatory in mice *in vivo*.

## 1 Introduction

The human pentraxin proteins, serum amyloid P component (SAP) (Pepys et al., 1997) and C‐reactive protein (CRP) (Pepys and Hirschfield, 2003), are normal circulating plasma proteins which are important in routine clinical diagnosis. They are also targets for novel therapies currently being developed for major diseases (Pepys et al., 2002, 2006; Kolstoe et al., 2009; Bodin et al., 2010; Gillmore et al., 2010). However some of their putative roles in health and disease are controversial. Comprehensively validated, highly purified, authentic, native, structurally intact and fully functional human SAP, isolated from normal human plasma, is essential for SAP scintigraphy (Hawkins et al., 1988b, 1990a, 1990b) in patients with amyloidosis (Pepys, 2006). Human CRP of comparable quality and authenticity is also necessary, for both critical experimental studies *in vitro* and *in vivo* studies in normal human volunteers, to rigorously establish its functional effects. Material for human use *in vivo* must be of pharmaceutical quality, produced under conditions compliant with current standards of current good manufacturing practice (cGMP), in order to be acceptable to ethical and regulatory authorities. We report here the production and characterization of the first such preparations.

Human SAP is universally present in all amyloid deposits (Pepys et al., 1997) as a result of its avid calcium dependent binding to all types of amyloid fibrils (Pepys et al., 1979b), regardless of their protein composition. We utilized this property in our invention of radiolabeled SAP scintigraphy for the safe, non‐invasive diagnosis and monitoring of amyloid deposits in systemic amyloidosis (Caspi et al., 1987; Hawkins et al., 1988b, 1990a, 1990b). This method revealed much of the previously obscure natural history of the different forms of systemic amyloidosis, including the critical fact that amyloid deposits can regress when the abundance of the respective amyloid fibril precursor protein is substantially reduced (Hawkins et al., 1993a, 1993b; Holmgren et al., 1993; Hawkins, 1994). These observations have underpinned major advances in diagnosis and management of amyloidosis and led to much improved patient survival, especially in the UK National Health Service National Amyloidosis Centre which is directly funded by the UK Department of Health to provide diagnostic and management advisory services for the whole UK national caseload. The Centre follows the largest and most diverse cohort of systemic amyloidosis patients in the world, currently sees more than 3000 patients and performs about 1000 SAP scintigraphy examinations annually. The investigation requires intact, native, highly purified, clinical GMP grade human SAP for labeling with ^123^I and intravenous injection into the patient. Unrelated to its use in diagnosis and monitoring, SAP contributes to the pathogenesis and/or persistence of amyloid deposition *in vivo* and is the target of novel therapeutic approaches to elimination of amyloid deposits which we have invented and are developing for clinical testing in collaboration with GlaxoSmithKline (www.pentraxin.com) (Pepys et al., 2002; Kolstoe et al., 2009; Bodin et al., 2010; Gillmore et al., 2010).

The physiological functions of neither human SAP nor CRP are fully understood but the most robust and reproducible observations indicate that they contribute to innate immunity against some bacterial infections. We have demonstrated this for smooth Gram negative bacteria with SAP (Noursadeghi et al., 2000) and for pneumococci with CRP (Loeffler et al., 2009). The avid binding of SAP to DNA (Pepys and Butler, 1987) and chromatin (Butler et al., 1990) strongly suggests that SAP may play a role in the appropriate, safe handling of these materials *in vivo*. More controversially it has been reported that SAP has an anti‐fibrotic effect, for which several different mechanisms have been claimed, most recently via stimulation of IL‐10 production (Castaño et al., 2009). There is even more wide ranging controversy over possible biological roles of human CRP, which has been claimed to be pro‐inflammatory, cytokine stimulating, pro‐atherogenic and pro‐thrombotic (Ballou and Lozanski, 1992; de Maat and Trion, 2004; Labarrere and Zaloga, 2004; Bisoendial et al., 2005, 2007a, 2007b, 2009). However human SAP is a constitutive plasma protein with a circulating concentration in the range of about 20-50 mg/L (Nelson et al., 1991) which is tightly regulated and almost constant in each individual. In contrast, human CRP is the classical, highly dynamic, rapidly responsive, entirely non‐specific acute phase protein with a 10,000 fold concentration range of about 0.05 to over 500 mg/L (Shine et al., 1981; Pepys and Hirschfield, 2003). Neither of these behaviors is consistent with a role in regulation of cytokine production and there is absolutely no clinical evidence in humans or experimental evidence in animals that endogenously produced high human CRP concentrations are inherently pro‐inflammatory. There are also compelling, well controlled, rigorous *in vitro* and *in vivo* studies which show no stimulation of cytokine production by the pentraxins (Hirschfield et al., 2003, 2005; Gillmore et al., 2004; Pepys, 2005; Pepys et al., 2005; Taylor et al., 2005; Taylor and van den Berg, 2007; Tennent et al., 2008).

Most reports on pro‐inflammatory effects of human CRP preparations have used inadequately characterized material isolated from human biological fluids or, more recently, commercial recombinant CRP produced in *E. coli*. The latter, manufactured only by the Oriental Yeast Company of Japan (Tanaka et al., 2002), is intended for use as an immunochemistry standard, and is sold by many different biochemical reagent companies. It is heavily contaminated with endotoxin and likely other bacterial products (Pepys et al., 2005). Although it has been claimed that a single gel filtration step removed all such contamination from this recombinant product (Bisoendial et al., 2005), experiments in two independent laboratories, using authentic, highly purified, very low endotoxin content, human CRP did not produce any pro‐inflammatory effects *in vitro* or *in vivo* in mice (Pepys et al., 2005; Taylor et al., 2005). The reports claiming anti‐fibrotic activity of SAP are also poorly controlled and/or otherwise flawed (Pilling et al., 2003; Haudek et al., 2006; Pepys et al., 2007; Tennent et al., 2007). Their conclusions are not supported by the complete absence of any abnormalities of connective tissue or fibrosis in patients on long term treatment with the SAP‐depleting drug, CPHPC, in whom SAP values are persistently reduced by 90-99% (Gillmore et al., 2010), or in mice with either deletion of the SAP gene or transgenic expression of human SAP (Botto et al., 1997; Bickerstaff et al., 1999; Gillmore et al., 2004). In order to provide suitable reagents with which to resolve these various controversies we have isolated from the plasma of healthy individuals, pharmaceutical GMP grade preparations of human CRP and SAP and fully characterized them as contaminant‐free and structurally and functionally intact.

## 2 Materials and methods

### 2.1 Plasma collection and testing by the Bio Products Laboratory Ltd

Plasma, derived exclusively from paid donors in the USA, was collected at centers approved by the UK Department of Health. Donor selection, donor examination and plasma collection were performed according to standards and/or requirements set by the UK Department of Health, in accordance with the European Pharmacopoeia monograph ‘Human Plasma for Fractionation’. Every donation was tested and found non‐reactive for: i) hepatitis B surface antigen (HBsAg); ii) antibodies to hepatitis C virus (HCV); iii) antibodies to human immunodeficiency virus 1 and 2 (HIV); iv) hepatitis A virus, HIV, HBV, HCV and parvovirus B19 by nucleic acid amplification technique (NAT) conducted by minipool testing. Units with a parvovirus B19 titer of greater than ~ 10^5^ IU/mL were excluded to guarantee that the B19 titer of the starting pool did not exceed 10^4^ IU/mL. Arrangements for plasma pool testing complied with the requirements of the CPMP Note for Guidance on Plasma‐Derived Medicinal Products CPMP/BWP/269/95. The plasma pool used for the preparation was derived from thousands of individual donors and was tested by the Bio Products Laboratory Ltd (BPL) and by the UK National Institute for Biological Standards and Control (NIBSC). Tests for HBsAg, anti‐HIV1/2 and anti‐HCV and for HCV RNA by NAT were negative (non‐reactive) for all tests. Arrangements for manufacturing complied with the requirements of CPMP Note for Guidance on Plasma‐Derived Medicinal Products CPMP/BWP/269/95. The standard operating procedure covering the donations details the actions to be taken in the case of a known or suspected defect of a donation and includes notifying any third party supplied with this material.

### 2.2 Isolation of SAP and CRP from plasma

The starting pool of plasma, collected by plasmaphoresis using sodium citrate anticoagulant, was stored at -35 °C, before conditioning at -10 °C for ~ 50 h and then thawed at ~ 0 °C to + 2 °C for collection of the cryoprecipitate by centrifugation. The supernatant was treated with 0.5% w/w celite (Hyflo Supercel) before ethanol fractionation based on a modification of the Kistler and Nitschmann method (Kistler and Nitschmann, 1962). Fraction A + 1 was precipitated at pH 5.85, 19% v/v ethanol and -5 °C and collected by centrifugation. This paste was resuspended in water for injection, the ionic strength adjusted using sodium phosphate/acetic acid buffers (Kistler and Nitschmann, 1962) and fraction B + 1 was precipitated at pH 5.1, 17% v/v ethanol and -3 °C and collected by centrifugation. The B + 1 paste contains SAP and CRP at about 500-900 mg/kg and 10-20 mg/kg respectively, reflecting their respective concentrations in normal human plasma of about 20-40 mg/L and 0.8 mg/L. The pentraxins were isolated from 38 kg of B + 1 paste by solubilization in 10 mM Trometamol, 140 mM NaCl, 1 mM EDTA, pH 8.0, fractionation on DEAE Sephadex and then calcium dependent affinity chromatography on phosphoethanolamine covalently immobilized on Sepharose, as previously described (Pontet et al., 1978; de Beer and Pepys, 1982; Hawkins et al., 1991; Carlucci et al., 2010). Briefly, the extracted B + 1 paste was depth filtered on a Millipore CE15 filter before adding 5% v/v of 0.2 M EDTA, pH 7.0 and mixing 437 kg of the solution with 6 kg of dry DEAE Sephadex which had been swollen in distilled water and then equilibrated with 10 mM Trometamol, 140 mM NaCl, 1 mM EDTA, pH 8.0, making a wet weight of gel of ~ 100 kg. After 1 h at room temperature the DEAE was washed with 10 mM Trometamol, 140 mM NaCl, 1 mM EDTA, pH 8.0, to remove unbound proteins before eluting the bound proteins with 2 M NaCl. All these steps were conducted at 8-15 °C. Trometamol (100 mM) and CaCl_2_ (50 mM) solutions were added to the eluate to yield a final concentration of 10 mM Trometamol, 5 mM CaCl_2_ at pH 8.0 before sequential filtration at 20 °C through a Pall Preflow UB filter followed by a Pall Flurodyne II 0.45 μ filter (Pall Corporation). The filtrate was then subjected to solvent‐detergent treatment with polysorbate 20 (8.8 g/L) and tri‐n‐butyl phosphate (2.45 g/L) for 120 min at 22-26 °C. This virus inactivation procedure was prospectively validated using HIV and independently audited. The process was also concurrently validated using three other enveloped viruses: sindbis, bovine viral diarrhea virus (BVDV) and infectious bovine rhinotracheitis virus (IBRV). The reductions in virus titers achieved were > 5.3 logs for HIV, > 7.0 logs for sindbis, > 4.0 logs for BVDV and > 6.4 logs for IBRV, providing good assurance that the solvent‐detergent step would be effective against HIV1/2 and HCV if they were present. There is no universally accepted model for HBV, but solvent detergent is also expected to be very effective against this lipid‐enveloped virus. The 414 kg eluate from DEAE was then mixed with 7 L of phosphoethanolamine-Sepharose which was synthesized using NHS‐activated Sepharose Fast Flow according to the manufacturer's instructions (GE Healthcare). After 2.5 h at room temperature to enable the SAP and CRP to bind to the immobilized phosphoethanolamine, the fluids were removed by filtration and the resin was washed with 10 mM Trometamol, 140 mM NaCl, 2 mM CaCl_2_, pH 8.0 until no further protein eluted. The beads were then poured into a BPG 200/500 chromatography column (GE Healthcare), packed with the same wash buffer, and the bound CRP was eluted with 1 mM phosphocholine in solution in the same buffer. The eluted CRP was collected, pooled in sterile plastic bags and stored frozen at ≤-35 °C. SAP bound to the column was then eluted with 10 mM Tris, 140 mM NaCl, pH 8.0 containing 10 mM EDTA and stored frozen at ≤-35 °C. The two non‐enveloped viruses which have been associated with disease transmitted by transfusion of blood and blood products, hepatitis A and human parvovirus B19, are not affected by solvent‐detergent procedures. The eluates containing SAP and CRP from the phosphoethanolamine-Sepharose column were therefore filtered through Pall DV50 50 nm and Pall DV 20 20 nm filters respectively, to reduce the risk from these viruses. The integrity of the nm filters was validated after use. At the time of the SAP preparation in 2004-5, 50 nm filtration had been approved for several product lines but when the CRP was filtered in 2008, 20 nm filtration, which is more effective against B19, was in more common usage. The filtered SAP was concentrated and then buffer exchanged against 10 volumes of 10 mM Trometamol, 140 mM NaCl, pH 8.0 on a Millipore Pellicon device with a 30,000 Da cut off membrane, to remove EDTA and any remaining traces of polysorbate 20 and tri‐n‐butyl phosphate. EDTA, 0.2 M pH 7.0, was added to the virus filtered CRP to a final concentration of 10 mM to chelate calcium and release bound phosphocholine before concentration and buffer exchange against 10 volumes of 10 mM Tris, 140 mM NaCl, pH 8.0 to remove all phosphocholine, EDTA and any remaining traces of polysorbate 20 and tri‐n‐butyl phosphate. After harvesting the concentrated CRP, 1 M CaCl_2_ was added to provide a final calcium concentration of 2 mM. Finally the isolated proteins were pre‐filtered at 1.2 μm and then sterile filtered at 0.22 μm into sterile containers. The suitably aliquoted preparations were stored, CRP at 3 mg/mL at 4 °C and SAP at 15 mg/mL frozen at -80 °C. All buffers and solutions were made up in sterile water for injection and the whole isolation was conducted under strict pharmaceutical GLP conditions and was fully compliant with GMP.

### 2.3 Characterization of the isolated GMP CRP and SAP preparations

The concentrations of salt, buffer salts and residual solvent detergent materials were assayed by standard methods. Total protein concentration was determined, in triplicate samples diluted with their respective solvents to produce absorbance values of about 0.1 with a 1 cm light path, by measuring net *A*_280_ after subtraction of *A*_320_ produced by light scattering. The precisely measured specific extinction coefficients (1% w/v, 1 cm) of 17.1 for human SAP and 17.5 for human CRP (de Beer and Pepys, 1982) were used to calculate the respective protein concentrations. Bacterial endotoxin was assayed by the kinetic LAL test, strictly according to the European Pharmacopoeia Monograph for Bacterial Endotoxins 2.6.14, and bacterial growth was sought by standard culture methods. Protein purity was assessed by SDS 8-18% PAGE (ExcelGel, GE Healthcare) heavily overloaded with samples run under reducing conditions and stained with Brilliant blue R350. Native protein integrity, absence of aggregation and dissociation were demonstrated by native, non‐denatured 3-8% gradient PAGE in Tris acetate (NuPAGE Novex, Invitrogen), and by size exclusion chromatography of 0.02 mg samples in a volume of 100 μL on a 10 × 30 cm Superdex 200 column equilibrated and eluted at 0.5 mL/min with 10 mM Tris, 140 mM NaCl, pH 8.0 for SAP and 10 mM Tris, 140 mM NaCl, 2 mM CaCl_2_, pH 8.0 for CRP. The integrity of the protomers of SAP and CRP was verified by electrospray ionization mass spectrometry (ESIMS). After buffer exchange into pure water 2-4 μL samples were diluted 1/10 with a 50%MeCN/49.9%H_2_O/0.1%HCOOH v/v/v mixture and infused into the electrospray source of a Quattro II triple quadrupole mass spectrometer (Micromass) under the following conditions: ES positive ion mode, 2.49 s scan with 0.11 s interscan delay, mass range *m/*z700–2750, cone voltage ramp 17–116 V, capillary at 3 kV. The concentrations of the specific proteins were confirmed by specific immunoassays for human CRP (Eda et al., 1998; Erlandsen and Randers, 2000) and SAP (Nelson et al., 1991) respectively. Functional integrity of the proteins for specific ligand recognition *in vitro* was established by their complete, strictly calcium dependent binding to phosphoethanolamine-Sepharose (Hawkins et al., 1991). The authentic native state of the SAP preparation and its functional integrity for localization to amyloid deposits were investigated *in vivo* in normal healthy C57BL/6 mice and C57BL/6 mice in which AA amyloidosis had been induced by repeated injection of casein (Hawkins et al., 1988a, 1991), in comparison with a highly purified non‐GMP batch of human SAP. SAP was trace radiolabeled with ^125^I as previously described (Hawkins et al., 1988a, 1991). Unlabeled non‐GMP SAP was spiked with labeled GMP SAP at approximately 0.3 μg (100,000 cpm) per mg. Normal healthy adult female C57BL/6 mice received 1 mg of the spiked SAP by IV injection and were then bled at intervals thereafter for assay of total human SAP by electroimmunoassay and counting to estimate clearance of the labeled GMP human SAP. Two groups of AA amyloidotic mice received 0.3 μg tracer doses of either GMP or non‐GMP ^125^I‐SAP by IV injection. After 24 h they were bled out, killed and radioactivity was determined in the spleen and liver, which contain the amyloid deposits in this model. Pro‐inflammatory effects of the preparations *in vivo* were sought in wild type adult female C57BL/6 mice weighing ~ 20 g each, which were pre‐bled 48 h before testing to provide individual baseline values of the sensitive murine acute phase reactants, SAP (Pepys et al., 1979a) and serum amyloid A protein (SAA), and then given 720 μg per mouse of each human protein IV (~ 30 mg/kg). Control mice received vehicle alone and all were bled out at 24 h for assay of mouse SAP (Pepys, 1979) and SAA (BioSource UK) in the serum.

### 2.4 Effects of the GMP SAP and CRP preparations on cytokine release by human peripheral blood mononuclear cells *in vitro*

*Assay reagents*. Aseptic technique was used for antibody manipulations and for the cell culture procedures. Antibodies and reagents for cell culture procedures were free from detectable pyrogen/endotoxin. Culture medium for all experiments was MEM (Gibco 21090) supplemented with 2 mM l‐glutamine (Sigma G7513), 100 U/mL penicillin and 0.1 mg/mL streptomycin (Sigma P0781), non‐essential amino acids (Gibco 11140), and 1 mM HEPES (Sigma H0887). Phosphate buffered saline (PBS) was prepared by dilution of sterile 10x stock solution (without calcium and magnesium, Gibco 70011-036) with sterile water (Baxter UKF7114). Dilutions of proteins and endotoxin were tested in quadruplicate with cells from four donors in each assay. *Isolation of peripheral blood mononuclear cells (PBMCs)*. Human whole blood was donated by consenting employees of NIBSC in accordance with local ethical practice. Donors were healthy males and females aged mid twenties to mid sixties, free of symptomatic viral and bacterial infections and who had not taken steroid anti-inflammatory medicines during the previous 7 days or non‐steroid anti‐inflammatory medicines during the 3 days prior to giving blood, nor were taking any other drug known to influence immunological responses. PBMCs and donor plasma were isolated, within 30 min of venesection, from heparinized (Fragmin Dalteparin Sodium, Pharmacia, 10 IU/mL blood) whole blood by density gradient centrifugation using Histopaque-1077 (Sigma H8889) layered beneath whole blood diluted 1/2 with PBS. Centrifugation at 340 *g* was used to separate PBMCs and plasma at room temperature and for washing the cells. After washing 2-3 times in PBS and re‐suspension in culture medium, PBMCs were stored in a humidified incubator at 37 °C, 5% CO_2,_ and used within 5 h of venesection. Donor plasma was stored at room temperature until used, also within 5 h of venesection. *Enzyme linked immunosorbent assay (ELISA) for cytokines*. ELISAs for the measurement of TNFα, IL‐6 and IL‐8 were carried out as previously described (Findlay et al., 2010). WHO international standards (IS) produced at NIBSC for TNFα, IL‐6 and IL‐8 were used as calibrants for the cytokine ELISAs (preparation 88/786 for TNFα, 89/548 for IL‐6 and 89/520 for IL‐8). The standards, two‐fold dilutions ranging from 15.6 to 4000 pg/mL, were diluted in cell culture medium supplemented with 2% v/v plasma. Supplemented culture medium was used as a blank. For the measurement of IL‐1β, monoclonal anti‐human IL‐1β capture antibody (Duoset DY201, R & D Systems) was added in PBS, to wells of 96‐well microtiter plates (Immuno MaxiSorp, NUNC) at 1 μg/mL (100 μL/well). Plates were covered and left for 16-24 h at 4 °C prior to washing 3 times with wash buffer (PBS containing 0.1% v/v Tween 20, Fisher Scientific). Plates were blocked with 100 μL of 1% w/v bovine serum albumin (Sigma A7888) in PBS. Cell‐conditioned medium, 50 μL, or IL‐1β standard (WHO IS 86/680, NIBSC) at 2‐fold dilutions ranging from 15.6 to 4000 pg/mL (in culture medium containing 2% v/v plasma or serum as specified below), was added to each well coated with capture antibody. Concentrations of standard and supplemented culture medium alone were added to every microtiter plate in duplicate. Biotinylated polyclonal anti‐human IL‐1β detection antibody (Duoset DY201, R & D Systems) 50 μL in PBS containing 1% w/v bovine serum albumin was added to wells prior to an overnight incubation of the covered plates at 4 °C. Plates were washed 3 times in wash buffer prior to addition of 100 μL peroxidase‐conjugated streptavidin (Jackson ImmunoResearch Laboratories) in wash buffer; plates were incubated for 15 min at room temperature and then washed 3 times in wash buffer and once in demineralized water. O‐phenylenediamine dihydrochloride substrate solution (Sigma P8787), 100 μL in citric‐acid monohydrate solution containing 30% v/v hydrogen peroxide, was added and, 5-10 min later, 50 μL 1 M sulfuric acid. The absorbance values were calculated by subtracting the OD values measured using a corrective 540 nm filter from the OD values measured with a 450 nm filter. ELISA of IL‐10 was as for IL‐1β except that IL‐10 Duoset DY217B (R & D Systems) was used and the IL‐10 standard was WHO IS 93/772, NIBSC. *Cytokine release studies using human PBMC (monocyte activation test described in the European Pharmacopoeia 2.6.30)* were conducted as described previously (Poole et al., 2003; Gaines Das et al., 2004). Briefly, PBMC were isolated from human heparinized peripheral blood within 4 h after its collection as described above. Clinical grade CRP and SAP proteins were incubated with 0.5–1.0 × 10^6^ PBMC/mL in 250 μL of supplemented MEM culture medium containing 2% v/v autologous plasma. All cultures were in quadruplicate under aseptic conditions, with sterile, pyrogen free reagents and consumables, at 37 °C, in 5% CO_2_ in humidified air for 16–24 h. All responses to CRP and SAP were compared with simultaneous responses to bacterial endotoxin (the second WHO international endotoxin standard, 94/580) in the same assays, including spiking experiments.

## 3 Results

### 3.1 Protein purity

**Fig. 1:**
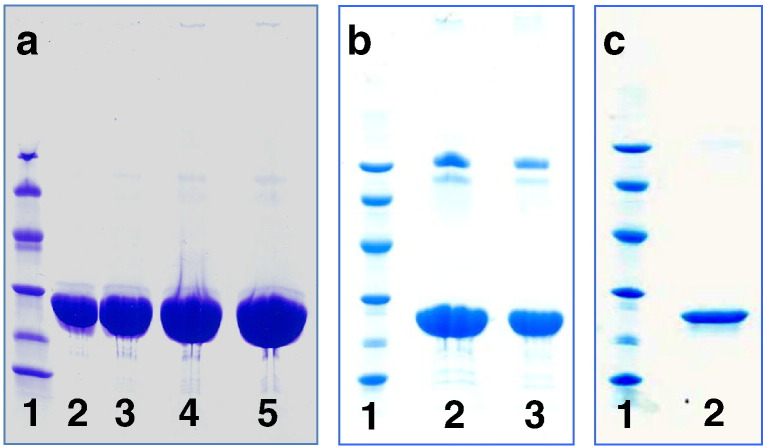
SDS 8-18% PAGE of reduced samples of GMP human pentraxins. a, GMP human SAP. Lane 1, molecular weight marker proteins: 94 kD, 67 kD, 43 kD, 30 kD, 20.1 kD, 14.4 kD; lane 2, SAP 25 μg; lane 3, SAP 50 μg; lane 4, SAP 75 μg; lane 5, SAP 100 μg. b, GMP human CRP. Lane 1, marker proteins; lane 2, CRP 60 μg; lane 3, CRP 30 μg. The trace higher molecular weight impurities identified by proteomic analysis as IgM μ chain and plasmin/plasminogen respectively are seen in lanes 2 and 3. c, GMP human CRP. Lane 1, marker proteins; lane 2, CRP 5 μg.

The isolated SAP preparation at 15 mg/mL contained 6 mg/L residual polysorbate‐20 and < 0.2 mg/L of tri‐n‐butyl phosphate. These compounds were not assayed in the final CRP preparation, which was at 3 mg/mL, but it had undergone the same extensive buffer exchange, ‘washing’ process, as the SAP. Both protein preparations were sterile with no bacterial growth on culture. The bacterial endotoxin content of the SAP was < 0.003 EU/mg and for CRP was < 0.1 EU/mg, that is below the detection limit detection with the CRP at 3 mg/mL. Heavily overloaded SDS-PAGE of the SAP preparation showed no significant bands other than SAP itself (Fig. 1a). The very faint bands seen in lanes loaded with more than 50 μg of SA comprise less than 0.1% of the total protein. The CRP preparation contained two very faint bands on either side of the 94 kD marker, in addition to CRP (Fig. 1b). These other proteins comprised less than 1% of the total protein and their presence is not surprising because several chromatographic steps required to remove traces of other proteins in preparing the most highly purified CRP (de Beer and Pepys, 1982; Hawkins et al., 1991; Carlucci et al., 2010) were not possible in the present pharmaceutical GMP procedure. The trace higher molecular weight proteins were identified by proteomic analysis, using mass spectrometry of trypsin digested fragments of the bands excised from the SDS‐PAGE. The lower mass band was the μ heavy chain of IgM and the higher mass band was plasmin/plasminogen (data not shown). The very faint trace bands of mass lower than the SAP and CRP protomers are characteristic cleaved fragments of the protomers which are derived from intact pentameric pentraxins when they are reduced and denatured; they are invariably present in pentraxins isolated from *ex vivo* human material. No SAP was detected in the CRP preparation and no CRP was detected in the SAP preparation, which were tested at 3.0 and 1.5 mg/mL respectively using assays which in both cases detected the other pentraxin at 1 μg/mL (Nelson et al., 1991; Shine et al., 1981).

### 3.2 Protein structural integrity

**Fig. 2:**
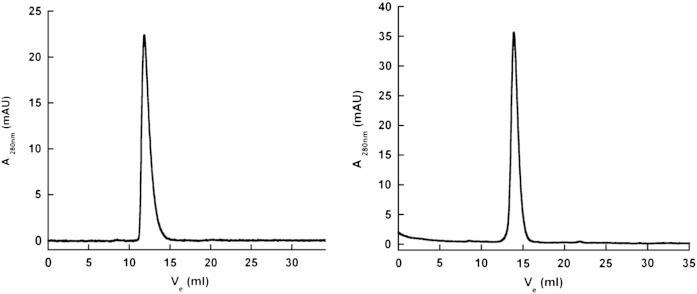
Size exclusion chromatography of GMP human pentraxins on Superdex 200. Both SAP (left) and CRP (right) gave single sharp peaks corresponding to their known molecular masses under the conditions used, in which SAP runs as stable decameric assemblies of pairs of the native pentameric structure and CRP runs as a single native pentamer.

**Fig. 3:**
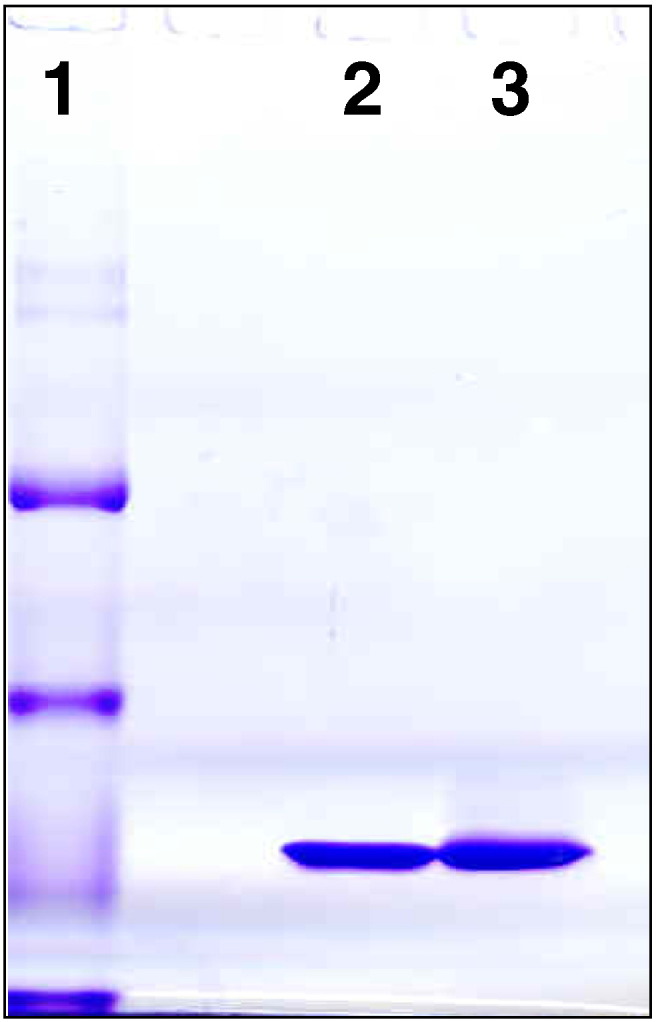
Native non‐denatured 3-18% gradient PAGE of human SAP. Lane 1, molecular weight marker proteins: 669 kD, 440 kD, 232 kD, 140 kD; lane 2, GMP human SAP 13 μg; lane 3, non‐GMP human SAP for comparison 13 μg. Human SAP is known to run as a stable decamer, mass 254,620, under these conditions (de Beer et al., 1982).

The authentic covalent structures of the protomers of each pentraxin were confirmed by ESIMS, with average molecular masses (SD) (n = 3) of 25,462.64 (0.39) for SAP and 23,027.46 (0.52) for CRP, corresponding exactly to the predicted masses for their respective amino acid sequences, plus glycan in the case of SAP (Pepys et al., 1994) and with *N*‐terminal PCA in CRP (Oliveira et al., 1979). Integrity of the authentic native non‐covalent pentameric assembly of each protein was confirmed by gel filtration chromatography, which also showed the absence of any aggregation or dissociation into free protomers (Fig. 2). The same result was obtained with the SAP preparation in non‐denatured gradient PAGE (Fig. 3). Unlike the 4-30% gradient gels in Tris glycine we have previously used (de Beer et al., 1982) but which are no longer available, human CRP does not form a discrete band in the present system.

### 3.3 Protein functional integrity

**Fig. 4:**
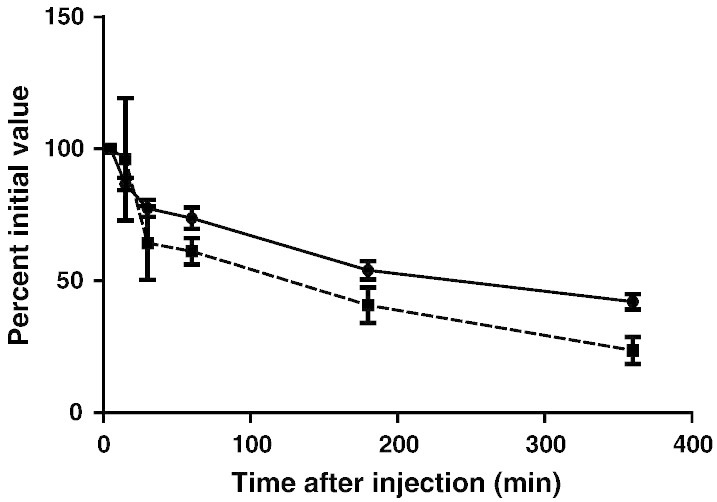
Blood clearance of tracer ^125^I‐GMP SAP (● ●) and abundant (1 mg/mouse) unlabeled non‐GMP SAP (■---■) administered simultaneously IV to adult C57BL/6 mice (n = 3). Each point represents the mean (SD) of measurements in 3 mice. The initial elimination of a larger proportion of the non‐GMP material probably reflects the presence of aggregated molecules not present in the GMP product but the subsequent clearance kinetics are essentially the same for the two preparations confirming that the labeled GMP SAP behaves like the intact native protein.

**Fig. 5:**
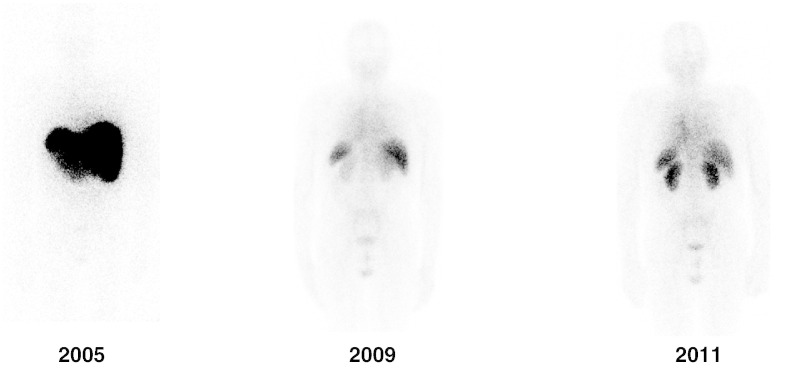
Posterior whole body images of SAP scintigraphy scans of a patient with systemic monoclonal immunoglobulin type (AL) amyloidosis who presented with major liver involvement and proteinuria in 2005. He responded well to chemotherapy with substantial regression of amyloid by 2009 when his liver and renal function had returned to normal. The underlying plasma cell dyscrasia then gradually relapsed during 2009-11 leading to recurrence of proteinuria caused by reaccumulation of renal amyloid shown in the 2011 scan.

Functional activity of the proteins was confirmed by their reproducible, 100%, strictly calcium dependent binding to phosphoethanolamine-Sepharose beads (not shown). Furthermore these human proteins had the expected plasma clearance half life of ~ 3-4 h after intravenous injection into normal wild type C57BL/6 mice (Baltz et al., 1985; Hawkins et al., 1988a) (shown for SAP in Fig. 4; not shown for CRP). This is a very sensitive test for structural and functional integrity of plasma proteins as even extremely subtle alterations, which may be undetectable by *in vitro* biophysical and biochemical methods, cause accelerated clearance of plasma proteins from the circulation *in vivo*. A key functional property of human SAP is its avid binding to amyloid deposits *in vivo* (Pepys et al., 1979b, 1997) and the clinical objective of the present GMP SAP preparation was to provide material for routine clinical SAP scintigraphy in the National Amyloidosis Centre. We therefore confirmed that trace radiolabeled GMP SAP was cleared in mice *in vivo* at precisely the same rate as a non‐GMP preparation of human SAP isolated in our laboratory (Fig. 4). Furthermore both the GMP and the non‐GMP SAP preparations localized to the same extent in the amyloidotic organs of mice with systemic AA amyloidosis. On this basis, we proceeded to use the GMP SAP for clinical scanning in patients with known or suspected amyloidosis and it has so far been deployed for this purpose in over 10,000 individuals with excellent results and no adverse effects whatsoever. Typical images are shown in Fig. 5.

### 3.4 Effect of GMP pentraxins on cytokine release *in vitro*

**Fig. 6:**
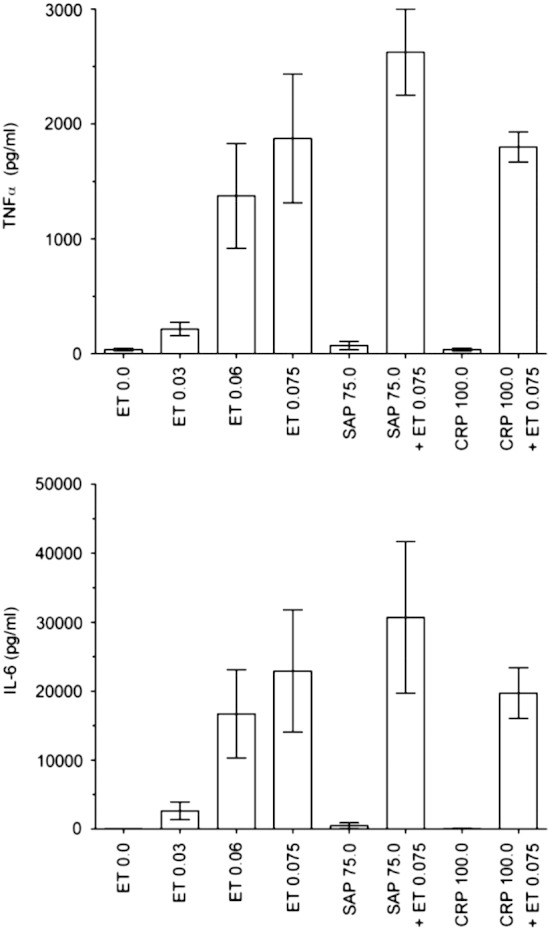
Absence of cytokine responses to hu man CRP and SAP. Bars show mean (SEM) of cytokine release by peripheral blood mononuclear cells from 4 individual donors cultured with endotoxin (ET, EU/mL), CRP or SAP (μg/mL).

**Fig. 7:**
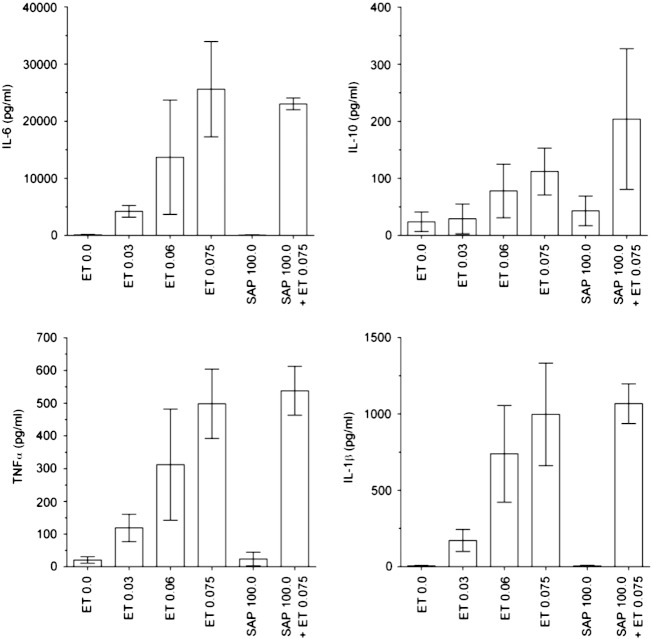
Absence of cytokine responses to hu man SAP. Bars show mean (SEM) of cytokine release by peripheral blood mononuclear cells from 4 individual donors cultured with endotoxin (ET, EU/mL) or SAP (μg/mL).

In four independent experiments (Tables 1, 2, Figs. 6, 7)) each using PBMC from four donors (15 different donors in total since one donor donated blood for both experiments 1 and 3), neither CRP (at up to 100 μg/mL with 11 donors in 3 independent experiments), nor SAP (at up to 100 μg/mL with 4 donors in one experiment and up to 75 μg/mL with 4 donors in one other experiment) stimulated release of TNFα, IL‐6, IL‐8, IL‐1β and IL‐10 above background values; IL‐1β and IL‐10 were measured only in response to SAP in experiment 4. In contrast, endotoxin stimulated dose‐dependent cytokine release from the PBMC of all donors (Tables 1, 2). CRP and SAP did not significantly enhance endotoxin mediated cytokine release, nor did they interfere in any of the cytokine assays; even at 100 μg/mL of each pentraxin, the assays gave endotoxin spike recoveries of 86-182% (Tables 1, 2).

### 3.5 Effect of GMP pentraxins on the in vivo acute phase response in mice

The murine acute phase proteins, SAP and SAA, respond with exquisite sensitivity to endotoxin and all other toxic and pro‐inflammatory materials which have been tested (Pepys et al., 1979a, 2005; Pepys and Baltz, 1983; Poole et al., 1984, 1986). However very high dose, ~ 30 mg/kg, intravenous bolus injections of neither GMP SAP nor GMP CRP stimulated an acute response (Table 3). This is consistent with our extensive previous experience in mice and rats receiving even higher doses of highly purified non‐GMP pentraxin preparations which were free of endotoxin contamination. It is also consistent with the present finding that neither of the pentraxin preparations stimulated cytokine release by human peripheral blood mononuclear cells *in vitro*.

## 4 Discussion

The function of a human plasma protein in humans can be definitively established by studying individuals with genetic deficiency or abnormality of the protein, by investigating effects of a specific intervention which persistently depletes the protein in question, or, possibly, by administering a highly purified preparation of the intact isolated protein. There is no rigorous report of any deficiency or even structural polymorphism of either human CRP or SAP proteins. Thorough characterization of preparations of these proteins, whether isolated from different individuals or from pools of donors, has so far shown only the single protein sequences corresponding to their respective single functional genes (Vigushin et al., 1993; Pepys et al., 1994). The single typical biantennary *N*‐linked oligosaccharide of human SAP is also invariant (Pepys et al., 1994); human CRP is not glycosylated (Vigushin et al., 1993). Thus no genetic ‘experiment of Nature’ is available for the human pentraxins. Our drug CPHPC (Pepys et al., 2002) which produces persistent 90-99% depletion of circulating human SAP for as long as it is administered, has led to no functional deficit or detectable adverse effect in 31 adults with systemic amyloidosis treated for up to 7 years (Gillmore et al., 2010). Any role of human SAP must have been redundant in these individuals and in the 70 or so other adults subjected to SAP depletion so far, including healthy normal volunteers (unpublished), patients with Alzheimer's disease (Kolstoe et al., 2009) and patients with osteoarthritis (unpublished). No drug is yet available which depletes CRP although our novel bis‐phosphocholine compounds (Pepys et al., 2006) are in development for clinical testing (www.pentraxin.com).

The first GMP grade preparations of isolated human SAP and CRP which we report here are approved by the UK MHRA for administration respectively to patients and to human volunteers. SAP deficiency produces no abnormal phenotype in unchallenged mice, and since sustained almost complete SAP depletion in human patients with systemic amyloidosis, osteoarthritis or Alzheimer's disease has had no adverse effects, we consider it neither necessary nor ethical to investigate the effects of large doses of isolated human SAP in volunteers. Our use of pharmaceutical grade SAP is thus confined to routine clinical SAP scintigraphy in the National Amyloidosis Centre, where the dose is 50-100 μg per patient and over 15,000 studies have been conducted since 1988, including over 10,000 with the present GMP preparation, without any adverse effects.

In contrast, infusion of recombinant bacterial CRP, derived from material which is grossly contaminated with endotoxin (Pepys et al., 2005) and which was purified only by a single gel chromatography procedure (Bisoendial et al., 2005), elicited a marked inflammatory reaction in healthy human volunteers and in patients (Bisoendial et al., 2005, 2007a, 2007b, 2009). The authors ascribed these effects to human CRP and construed them as support for a pro‐atherogenic role of CRP. Our studies with authentic, highly purified human CRP, isolated from humans rather than from recombinant bacteria, and with very low endotoxin content, had no pro‐inflammatory effects either on cells *in vitro* or in mice *in vivo* (Pepys et al., 2005). These robust findings seriously questioned the interpretation of experiments with recombinant bacterial CRP (Pepys et al., 2005). However we produced the present GMP grade human CRP from normal human blood donor plasma, processed under strict pharmaceutical conditions throughout, specifically in order to rigorously test in humans whether human CRP itself, rather than any possible contaminants, had pro‐inflammatory effects *in vivo*. That study, approved by the UK MHRA, is currently in progress and will be reported separately. Meanwhile we tested both our GMP SAP and CRP preparations *in vitro* on human peripheral blood mononuclear cells and by injection into mice *in vivo* to determine whether they stimulated cytokine production and had pro‐inflammatory actions.

As shown here, neither protein preparation had any significant effect either on human mononuclear cells in culture *in vitro* or in mice *in vivo*. In particular human SAP did not stimulate production of IL‐10 and human CRP did not stimulate production of the pro‐inflammatory cytokines IL‐1, IL‐6 or TNFα. The compelling nature of these negative findings is robustly strengthened by the exhaustive demonstration that the proteins being tested were both structurally and functionally intact and contained no significant detectable contamination with endotoxin. Comparably rigorous sourcing of starting material, processing, purification and final product characterization of human CRP and SAP preparations are all essential before different or additional properties can credibly be assigned to these proteins. Our negative experimental observations with GMP human CRP are entirely consistent with the compelling experimental results which show that CRP either has no effect or may actually be anti‐atherogenic in animal models (Hirschfield et al., 2005; Kovacs et al., 2007; Tennent et al., 2008; Koike et al., 2009; Teupser et al., 2011). Finally there is also now overwhelming clinical epidemiological evidence that provides no support for a pro‐atherogenic role of human CRP (Emerging Risk Factors Collaboration et al., 2010; C Reactive Protein Coronary Heart Disease Genetics Collaboration (CCGC) et al., 2011).

## Figures and Tables

**Table 1:** Effect of GMP human pentraxins on cytokine production/release by human peripheral blood mononuclear cells *in vitro*.

Experiment	Protein	Cytokine responses		
		IL-6 (pg/mL)	TNFα (pg/mL)	IL-8 (pg/mL)
1	Medium only control	119 ± 46		
	CRP (1.0, 10.0, 100 μg/mL)	73 ± 33, 136 ± 28, 36 ± 21		
	ET (0.03, 0.06, 0.075 EU/mL)	1123 ± 725, 16207 ± 8710, 58204 ± 38546		
	ET spike recovery (%) for CRP (100 μg/mL) + ET (0.075 EU/mL):			
		95 ± 11%		
2	Medium only control	153 ± 16	238 ± 134	20365 ± 15185
	CRP (1.0, 10.0, 100 μg/mL)	133 ± 42, 277 ± 109, 81 ± 47	227 ± 103, 256 ± 135, 318 ± 159	13099 ± 17814, 27198 ± 20251, 31778 ± 12719
	ET (0.03, 0.06, 0.075 EU/mL)	1369 ± 438, 9595 ± 2180, 14675 ± 2549	652 ± 186, 2146 ± 203, 2007 ± 726	56188 ± 30074, 97784 ± 29648, 114944 ± 34071
	ET spike recovery (%) for CRP (100 μg/mL) + ET (0.075 EU/mL):			
		99 ± 11%	104 ± 4%	123 ± 29%
3	Medium only control	36 ± 29	37 ± 10	8098 ± 3554
	CRP (1.0, 10.0, 100 μg/mL)	43 ± 42, 71 ± 48, 56 ± 53	23 ± 7, 22 ± 6, 37 ± 12	7190 ± 2900, 7743 ± 3374, 10405 ± 4313
	ET (0.03, 0.06, 0.075 EU/mL )	2640 ± 1292, 16695 ± 6405, 22913 ± 8871	216 ± 59, 1374 ± 456, 1874 ± 560	62105 ± 22665, 141392 ± 38410, 159508 ± 45048
	ET spike recovery (%) for CRP (100 μg/mL) + ET (0.075 EU/mL):			
		86 ± 16%	96 ± 7%	88 ± 12%
3	Medium only control	36 ± 29	37 ± 10	8098 ± 3554
	SAP (0.75, 7.5, 75 μg/mL)	1 ± 1, 5 ± 4, 485 ± 422	24 ± 16, 22 ± 7, 72 ± 36	7126 ± 3859, 4981 ± 2432, 36270 ± 15847
	ET (0.03, 0.06, 0.075 EU/mL)	2640 ± 1292, 16695 ± 6405, 22913 ± 8871	216 ± 59, 1374 ± 456, 1874 ± 560	62105 ± 22665, 141392 ± 38410, 159508 ± 45048
	ET spike recovery (%) for SAP (75 μg/mL) + ET (0.075 EU/mL):			
		134 ± 48%	140 ± 20%	158 ± 19%
4	Medium only control	96 ± 78	21 ± 10	
	SAP (1.0, 10.0, 100 μg/mL)	24 ± 62, 66 ± 62, 72 ± 59	24 ± 9, 24 ± 18, 24 ± 21	
	ET (0.03, 0.06, 0.075 EU/mL)	4219 ± 1041, 13698 ± 10017*, 25589 ± 8349	119 ± 42, 312 ± 170, 498 ± 106	
	ET spike recovery (%) for SAP (100 μg/mL) + ET (0.075 EU/mL):			
		90 ± 4%*	108 ± 15%	

Values are mean ± SEM; n = 4 donors of PBMC except values for 3 donors indicated with an asterisk (*); ET, endotoxin.

**Table 2:** Effect of GMP human SAP on cytokine production/release by human peripheral blood mononuclear cells *in vitro*.

Experiment	Protein	Cytokine responses	
		IL-1β (pg/mL)	IL-10 (pg/mL)
4	Medium only control	6 ± 3	24 ± 17
	SAP (1.0, 10.0, 100 μg/mL)	24 ± 4, 24 ± 2, 5 ± 5	34 ± 20, 31 ± 18, 43 ± 26
	ET (0.03, 0.06, 0.075 EU/mL)	172 ± 72, 739 ± 316, 997 ± 335	29 ± 26, 78 ± 47, 112 ± 41
	ET spike recovery (%) for SAP (100 μg/mL) + ET (0.075 EU/mL):		
		107 ± 13%	182 ± 110%

Values are mean ± SEM; n = 4 donors of PBMC; ET, endotoxin.

**Table 3:** Effect of intravenous bolus injection of GMP human pentraxins on circulating concentrations of murine acute phase proteins.

Injection	Mouse SAA mean (SD) mg/L		Mouse SAP mean (SD) mg/L	
	Baseline	24 h post injection	Baseline	24 h post injection
Vehicle control n = 5	10 (3)	11 (3)	17 (19)	20 (21)
GMP SAP (~ 30 mg/kg) n = 5	9 (5)	12 (2)	12 (12)	18 (13)
Vehicle control n = 5	22 (1)	23 (3)	9 (1)	10 (2)
GMP CRP (~ 30 mg/kg) n = 5	24 (1)	24 (2)	9 (3)	12 (5)
